# Mapping the Dielectric Properties of Unknown Targets by Using a Network of Microwave Sensors: A Proof-of-Concept †

**DOI:** 10.3390/s19061270

**Published:** 2019-03-13

**Authors:** Claudio Estatico, Alessandro Fedeli, Gian Luigi Gragnani, Matteo Pastorino, Andrea Randazzo

**Affiliations:** 1 Department of Mathematics, University of Genoa, 16146 Genoa, Italy; estatico@dima.unige.it; 2 Department of Electrical, Electronic, Telecommunications Engineering, and Naval Architecture, University of Genoa, 16145 Genoa, Italy; alessandro.fedeli@unige.it (A.F.); matteo.pastorino@unige.it (M.P.); andrea.randazzo@unige.it (A.R.)

**Keywords:** sensor networks, microwaves, inverse scattering

## Abstract

The subject of this paper is the possible use of a network of microwave sensors to achieve a map of the electromagnetic properties of unknown targets. The basic idea is to use a set of microwave sensors to illuminate a region of interest and to measure the resulting axial component of the electric field. Measurements are then processed by means of a technique based on inverse-scattering, which provides an estimate map of the dielectric values of the area under examination, allowing to discriminate among possible targets. In order to initially evaluate the feasibility of the proposed approach, numerical results in a simulated environment are preliminarily considered and discussed. Furthermore, an initial test on experimental data in a simplified configuration is also presented.

## 1. Introduction

Wireless sensor networks (WSNs) are nowadays widely adopted for various purposes in different environments and application fields. Their initial developments can be traced back to several years, as testified by the interesting review in [1]. However, in recent times, there has been an increasing interest in the development of WSNs in more new areas [2,3,4,5], attesting to their ubiquitous role. The use of WSNs has had a further increase and acceleration in conjunction with the advent of the internet-of-things (IoT) [6,7].

In this framework, an interesting application is related to the possibility of retrieving information about the presence and properties of targets in the scenario in which the nodes are located [8,9,10]. Such a possibility stems from the fact that the propagation of the electromagnetic waves (which are generated by the sensors) is significantly affected by the distribution of the dielectric properties in the scenario, and thus, by the presence and by the geometrical/physical properties of the targets. Consequently, by properly processing the received signals, it is in principle possible to extract some information about the objects that perturbate the field, e.g., their presence and possibly their dielectric properties. To this end, two main research directions have been followed. The first one concerns the development of algorithms able to provide just the position of the eventually present targets. Approaches belonging to this class are usually based on the analysis of the time-of-flight [11,12], on the estimation of the directions of arrival of the impinging waves [13,14,15], or on the use of fingerprinting techniques relying on signal-strength measurements [16,17,18]. Despite their robustness and accuracy, these methods do not usually provide information about the type of target, its extent, and so on. Moreover, depending on the adopted algorithm, it may be difficult to identify the presence of more than one target without a-priori information. The second class of approaches is based on the use of imaging techniques aimed at providing an image representing some physical properties of the considered scenario. To this end, a possible solving strategy is to adopt electromagnetic inverse-scattering techniques, which are potentially able to retrieve the full distributions of the dielectric properties (dielectric permittivity and electric conductivity) starting from scattered-field measurements [19,20,21,22,23,24,25,26,27,28,29]. Differently from methods belonging to the first class, it is however necessary to adopt an exact model of the electromagnetic propagation inside the scenario. This is complicated by the fact that, from a mathematical point of view, the electromagnetic scattering phenomena are described by non-linear equations (with respect to the dielectric properties of the region). Moreover, the inverse problem of retrieving the dielectric distribution from electric field measurements is also strongly ill-posed. In order to address these issues, several inverse-scattering approaches have been proposed in the scientific literature. In particular, both qualitative and quantitative imaging techniques have been devised. In the first case, the retrieved image often represents an indicator function, which may give information about the positions, extents, and number of targets in the scene of interest. Some common qualitative approaches of this kind are the linear sampling method [30], the level set algorithm [31], and MUSIC [32]. Conversely, the images generated by quantitative methods also provide an estimation of the dielectric properties in any point of the inspected area, thus, allowing a characterization of the targets. Some examples of methods that belong to this class are the contrast source inversion technique [33] and various Newton-type schemes [34,35,36].

In this paper, which is an extended version of [37], the use of a new inverse-scattering technique belonging to the class of quantitative imaging methods is considered. Such a technique, adopted for producing images of the scenario in which the sensors are located, is preliminarily investigated by using numerical simulations in a simplified two-dimensional environment. Moreover, by employing the reference data provided by the Institut Fresnel [38], a simplified experimental scenario is also considered for preliminarily testing the developed procedure against real measurements. The inversion of the scattering model is performed by using a recently proposed technique developed in the framework of the $L^{p}$ Banach spaces [39,40,41], which is applied for the first time for addressing WSN imaging problems. In particular, a two-loop Newton-type scheme is adopted, in which the linearized problem obtained at each Newton step is solved in a regularized sense by using a truncated conjugate-gradient-like iterative algorithm in Banach spaces [42,43]. It has been found that such a class of approaches is able to address the non-linearity and the ill-posedness of the problem in an effective way. Furthermore, it is proven that in other imaging applications (e.g., medical and subsurface imaging) these methods are capable of providing better reconstruction performance than classical inversion techniques working in Hilbert spaces. In particular, the main advantages are related to the capabilities of $L^{p}$-based procedures (especially when the norm parameter *p* is lower than 2) of reducing the ringing and oversmoothing effects usually associated to the low-pass filtering that is usually introduced by standard regularization techniques. Such a reduction may allow to better reconstruct the shape and position of the targets, as well as to improve the estimation of their dielectric properties. Moreover, thanks to these advantages, small and localized objects can be retrieved with good accuracy.

The paper is organized as follows. In Section 2 the problem is formalized and a possible strategy for its solution is detailed. Some preliminary results, pertaining to an analysis carried out in a simplified 2D simulated environment, and an initial experimental validation in a simple test case, are presented in Section 3. Finally, in Section 4, conclusions are drawn.

## 2. Problem Description and Solution Strategy

**Figure 1:**
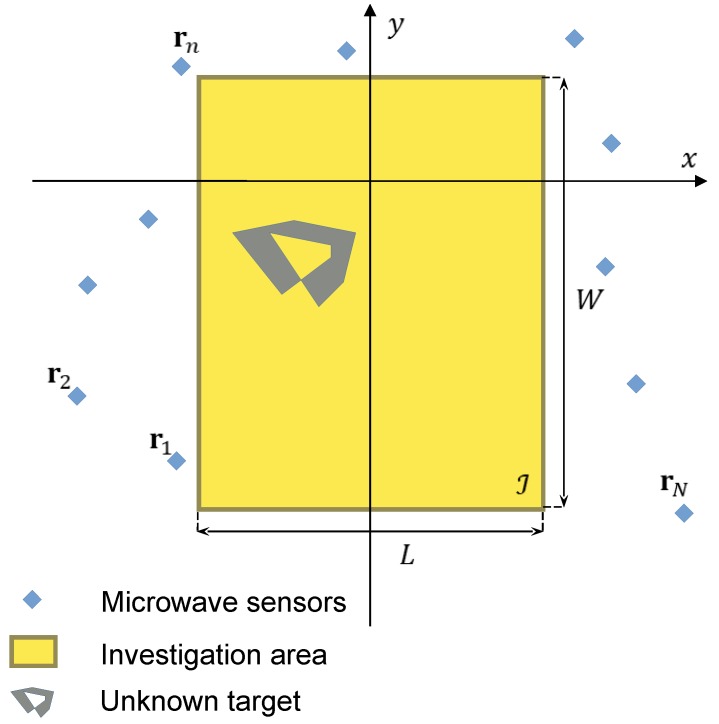
Microwave sensor network for characterizing dielectric targets: sketch of the adopted problem configuration.

The problem configuration is shown in Figure 1. A predefined region of space on the xy plane, denoted as the inspection domain I, contains some unknown targets. This region is surrounded by *N* microwave transceiver sensors, located in points $r_{n},\;n=1,\;\ldots ,\;N$. The background scenario is known, and a free-space configuration is considered. It is assumed that each sensor acts as a transceiver and can radiate or receive a time-harmonic electromagnetic field at a fixed frequency *f*. Furthermore, each sensor is able to collect measurements of the *z*-component of the electric field. The acquired field data are shared with an external control device, not discussed here, whose function is also to synchronize sensor measurements. During the acquisition phase, one device at a time operates in transmission mode, while all the other ones (i.e., N−1 elements) are employed for electric field measurements. Such a measurement process is repeated until all the *N* sensing elements have been used in transmission and reception modes.

With this set of field measurements available, the objective is to retrieve a map of the dielectric properties of the targets inside the inspection domain I. For the sake of simplicity, only a 2-D model, characterized by TM (with respect to the *z* axis) electromagnetic fields and object properties invariant along the same axis, is considered. In addition, non-magnetic materials (i.e., characterized by the same magnetic permeability as the vacuum, $\mu_{0}$) are assumed in this paper. However, extending the present formulation to a three-dimensional problem is, in principle, quite straightforward, even though the computer implementation can be challenging.

Basically, when an unknown target is present inside the investigation area, the electric field is perturbed with respect to the absence of objects. Consequently, this field perturbation (usually referred as the scattered electric field $E_{\mathrm{sca}}$), if properly measured, can be used to reconstruct the target properties [44]. In this work, we focus on retrieving a point-by-point map of the complex dielectric permittivity inside I, that is:

(1) $${\tilde{\epsilon }}_{r}\left(r\right)=\epsilon_{r}\left(r\right)-j\frac{\sigma \left(r\right)}{\omega \epsilon_{0}},\;\;r\in I.$$

In this equation, the term $\epsilon_{r}$ represents the real part of the relative dielectric permittivity, σ is the electric conductivity, ω=2πf is the angular frequency, and finally $\epsilon_{0}≃8.85\times 10^{-12}\;F/m$ stands for the vacuum dielectric permittivity.

In general, the scattered electric field $E_{\mathrm{sca}}$ in the *n*-th sensor location is related to the dielectric properties of the investigation area I by means of an integral equation, i.e.,

(2) $$E_{\mathrm{sca}}\left(r_{n}\right)=-k_{0}^{2}\int_{I}c\left(r\right)E_{t}\left(r\right)g\left(r_{n}|r\right)dr,\;n=1,\ldots ,N$$

where $k_{0}=\omega \sqrt{\mu_{0}\epsilon_{0}}$ is the vacuum wave number, $c\left(r\right)={\tilde{\epsilon }}_{r}\left(r\right)-1$ is the so-called *contrast function* (a free-space background, modeled as vacuum, is assumed), $E_{t}\left(r\right)$ is the total electric field (in the presence of the unknown targets), and *g* is the Green’s function of the considered configuration [44].

In order to implement such a model within a computer code, the inspection domain I is subdivided into *I* cells of square area $I_{i},\;i=1,\;\ldots ,\;I$, whose center is positioned at $r_{i}^{I}$, and where both the contrast function *c* and fields are supposed to be constant (in other words, the continuous model has been discretized by using piecewise-constant basis functions).

Assuming that the *m*-th sensor (positioned at $r_{m}$) is the only one in transmitting mode for the current view, and that, for each location of the transmitting device, the *z*-component of the scattered electric field $E_{\mathrm{sca}}$ is known at the positions of all the other N−1 sensors, the discrete version of (2) can be written as [39]

(3) $$E_{\mathrm{sca}}\left(r_{n}\right)=\sum_{i=1}^{I}c\left(r_{i}^{I}\right)E_{t}\left(r_{i}^{I}\right)h_{i}\left(r_{n}\right),\;\;n=1,\ldots ,N,\;\;n\neq m,$$

where the integral has been replaced by a summation, and the term $h_{i}$ is defined as

(4) $$h_{i}\left(r_{n}\right)=-k_{0}^{2}\int_{I_{i}}g\left(r_{n}|r\right)dr,$$

Equation (3) can be reformulated in a matrix form by introducing the following vectors:

(5) $$\begin{array}{c} c={\left[c\left(r_{1}^{I}\right),\;\ldots ,\;c\left(r_{I}^{I}\right)\right]}^{T},e_{t}={\left[E_{t}\left(r_{1}^{I}\right),\;\ldots ,\;E_{t}\left(r_{I}^{I}\right)\right]}^{T},\\ \;e_{\mathrm{sca}}={\left[E_{\mathrm{sca}}\left(r_{1}\right),\;\ldots ,E_{\mathrm{sca}}\left(r_{m-1}\right),E_{\mathrm{sca}}\left(r_{m+1}\right),\ldots ,E_{\mathrm{sca}}\left(r_{N}\right)\right]}^{T}, \end{array}$$

and it results that

(6) $$e_{\mathrm{sca}}=H_{\mathrm{dt}}\mathrm{diag}\left(c\right)e_{t}$$

The term $H_{\mathrm{dt}}$ is a rectangular matrix of size $\left(N-1\right)\times I$, and its elements are defined as ${\left[H_{\mathrm{dt}}\right]}_{n,i}=h_{i}\left(r_{n}\right)$. Actually, the total electric field in I in the presence of the targets is another unknown quantity, represented by the vector $e_{t}$, and it can be found with the aid of another equation (whose derivation is similar to the previous one), that is

(7) $$e_{t}=e_{\mathrm{in}}+H_{\mathrm{st}}\mathrm{diag}\left(c\right)e_{t}$$

in which the vector $e_{\mathrm{in}}={\left[E_{\mathrm{in}}\left(r_{1}^{I}\right),\;\ldots ,\;E_{\mathrm{in}}\left(r_{I}^{I}\right)\right]}^{T}$ contains the values of the electric field inside I without the unknown targets, and $H_{\mathrm{st}}$ is a square matrix of size *I* whose elements are given by ${\left[H_{\mathrm{st}}\right]}_{r,s}=h_{r}\left(r_{s}^{I}\right)$, with r,s=1,…,I.

By combining (6) and (7) together, we have:

(8) $$e_{\mathrm{sca}}=G\left(c\right)=H_{\mathrm{dt}}\mathrm{diag}\left(c\right){\left[I-H_{\mathrm{st}}\mathrm{diag}\left(c\right)\right]}^{-1}e_{\mathrm{in}}.$$

This equation represents a nonlinear relationship which links the contrast function values of the vector c to the measured scattered electric field values contained in $e_{\mathrm{sca}}$. Retrieving c starting from the knowledge of $e_{\mathrm{sca}}$ is a well-known inverse scattering problem. Unfortunately, due to the particular mathematical properties of this kind of problem, the previous equation turns out to be ill-posed. As a consequence, its solution is not trivial and should be accomplished with suitable inversion procedures, which should be capable of addressing the problem of ill-posedness as well as its non-linearity.

**Figure 2:**
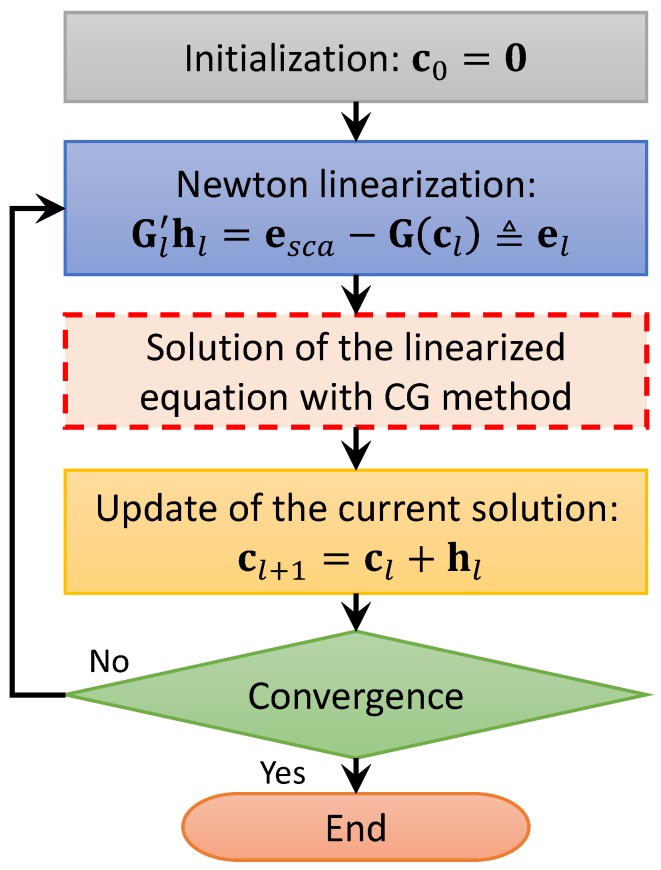
Flow-chart of the external loop of the algorithm employed for the quantitative characterization of dielectric targets.

The solution approach adopted in this work is an iterative deterministic scheme, based on a Newton-conjugate-gradient (NCG) method. In particular, this method operates in the mathematical framework of $L^{p}$ Banach spaces, and is composed by two nested iterative loops. In the external loop (whose iterations are indicated by the index *l*), an inexact-Newton approach is applied to linearize (8) around the value of the contrast function which is currently reconstructed, denoted as $c_{l}$. The main steps of the external loop are summarized in Figure 2.

**Figure 3:**
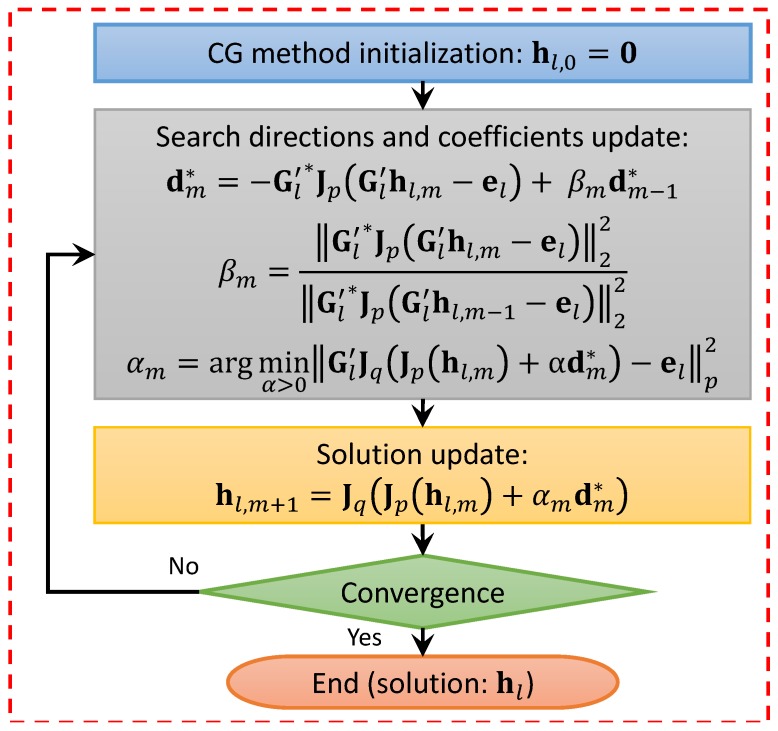
Flow-chart of the internal loop of the algorithm employed for the quantitative characterization of dielectric targets.

Once the problem is linearized, the resulting linear equation is solved in an inner loop by means of a regularizing non-conventional conjugate-gradient-like algorithm operating in $L^{p}$ Banach spaces, outlined in Figure 3 [43]. The parameter *p* can be tuned inside the inversion process, and is chosen as a fixed value before starting the iterations. Both the inner and the outer loops are terminated when the relative variation of the minimized residual functional falls below a predefined threshold.

In particular, the aim of the inversion method is to minimize the residual functional

(9) $$R_{p}\left(c\right)={∥G\left(c\right)-e_{\mathrm{sca}}∥}_{L^{p}}^{2}$$

where ${∥\cdot ∥}_{L^{p}}$ is the norm of the considered Banach space. Conversely, standard Hilbert-space regularization algorithms usually minimize the residual functional

(10) $$R_{2}\left(c\right)={∥G\left(c\right)-e_{\mathrm{sca}}∥}_{L^{2}}^{2}$$

and the application of the conjugate gradient method is straightforward. In $L^{p}$ Banach spaces, instead, this is no longer true, since the usual iteration scheme of the CG is not well defined. To overcome this problem, the method used in this paper is based on the concept of duality maps in Banach spaces, which, for the considered $L^{p}$ spaces, are defined as

(11) $$J_{p}\left(v\right)={∥v∥}_{L^{p}}^{2-p}{\left(\left|\upsilon_{1}\right|\mathrm{sign}\left(\upsilon_{1}\right),\ldots ,\left|\upsilon_{N}\right|\mathrm{sign}\left(\upsilon_{N}\right)\right)}^{T}$$

where $v=\left(\upsilon_{1},\ldots ,\upsilon_{N}\right)$ is a vector of *N* components, and

(12) $$\mathrm{sign}\left(\upsilon_{n}\right)=\left\{\begin{array}{cc} \exp\left(j\arg\left(\upsilon_{n}\right)\right) & \upsilon_{n}\neq 0\\ 0 & \mathrm{otherwise} \end{array}\right.$$

Note that, in usual Hilbert spaces with $L^{2}$ norm, the duality maps reduce to identity operators.

## 3. Preliminary Results

In order to assess capabilities and limitations of the method, different simulations have been performed. Furthermore, a very preliminary validation with experimental data has been carried out.

### 3.1. Simulated Environment

A simulated environment has been considered for the first proof-of-concept of the proposed characterization technique. A free-space scenario has been taken into account, where a network of microwave transceiving sensors operating at the frequency f=300MHz is located. The network is composed of N=15 elements. In turn, each of these sensors is used as a transmitter, whereas all the other ones (i.e., N−1 elements) have the function of measuring the scattered electric field resulting from the interactions between the incident wave and the targets in the inspection domain I. Since the method is based on registering the perturbation given by unknown targets with respect to a known background, an antenna with a non-directional pattern, able to collect from any spatial direction, is better suited than a directional one. Hence, in general, it is expected that very simple antennas, like dipoles, can work well. In the ideal 2D environment used for the simulation, this antenna behavior has been modeled by considering an infinite wire (line-current source), which provides an omnidirectional pattern in the transverse plane.

The forward electromagnetic simulations, which consist of computing the electric field data for a given dielectric configuration, have been carried out with a custom numerical code implementing a method-of-moment-based solver [45]. Moreover, in order to emulate more realistic operating conditions, the simulated field data have been corrupted with an additive white Gaussian noise with zero mean value and a signal-to-noise ratio equal to SNR=20dB.

A rectangular area with *x*- and *y*-directed sides of lengths L=1.6m and W=2.4m has been considered as the inspection domain I. This region is centered at coordinates x=0 and y=−2m. It is important to notice that, even though a rectangular area has been adopted here for the sake of simplicity, the developed approach can also deal with regions of arbitrary shape. For the solution of the forward problem, the domain I has been discretized into a mesh of $I_{f}=48\times 72$ square cells, in which each element has sides of length $d_{f}=0.033\;m$.

The target of the first test cases is represented by a cylindrical object with rectangular cross section, simulating the dielectric properties of dry wood and characterized by:

- center at the point $r_{c}=\left(0.2,\;-0.8\right)\;m$;
- side lengths $s_{x}=0.5\;m$ and $s_{y}=0.3\;m$;
- relative dielectric permittivity $\epsilon_{r}=3$;
- electric conductivity σ=0.01 S/m.

The parameters of the proposed Newton-conjugate-gradient characterization technique have been set as follows: fixed $L^{p}$ space exponent p=1.4, maximum inexact-Newton and conjugate gradient iterations M=50, threshold on the minimum relative variation of the residual cost function ΔR=0.15 in both loops. Furthermore, the inspection domain I has been subdivided into I=32×48 square cells with side length $d_{i}=0.05\;m$ for the inverse problem solution.

The performance of the proposed characterization technique has been evaluated by adopting some relative error parameters that measure the average difference between the actual and reconstructed dielectric properties in the background and the target regions, as well as in the whole inspection domain I. In more detail, these parameters are indicated as $\Gamma_{b}$, $\Gamma_{t}$, $\Gamma_{I}$, and are defined in this way:

(13) $$\Gamma_{b}=\frac{1}{I_{b}}\sum_{r_{i}\in I_{b}}\left|\frac{\tilde{\epsilon_{r}}\left(r_{i}\right)-\hat{\epsilon_{r}}\left(r_{i}\right)}{\hat{\epsilon_{r}}\left(r_{i}\right)}\right|,\;\;\Gamma_{t}=\frac{1}{I_{t}}\sum_{r_{i}\in I_{t}}\left|\frac{\tilde{\epsilon_{r}}\left(r_{i}\right)-\hat{\epsilon_{r}}\left(r_{i}\right)}{\hat{\epsilon_{r}}\left(r_{i}\right)}\right|,\;\;\Gamma_{I}=\frac{1}{I}\sum_{r_{i}\in I}\left|\frac{\tilde{\epsilon_{r}}\left(r_{i}\right)-\hat{\epsilon_{r}}\left(r_{i}\right)}{\hat{\epsilon_{r}}\left(r_{i}\right)}\right|,$$

where the reconstructed complex relative dielectric permittivity in the point $r_{i}$ is denoted as $\tilde{\epsilon_{r}}\left(r_{i}\right)$, and $\hat{\epsilon_{r}}\left(r_{i}\right)$ is the corresponding actual value in the same point. Moreover, the background and the target domains are represented by $I_{b},I_{t}$, and the numbers of cells contained inside them are equal to $I_{b}$ and $I_{t}$, respectively.

#### 3.1.1. Aligned Sensors

The first simulated scenario involves a network of aligned microwave sensors, positioned outside I along three sides of the domain characterized by y=0.5m, x=−1m, and x=1m, with 0.5 m spacing between each element. The detailed sensor locations in the xy plane are reported in Table 1.

**Figure 4:**
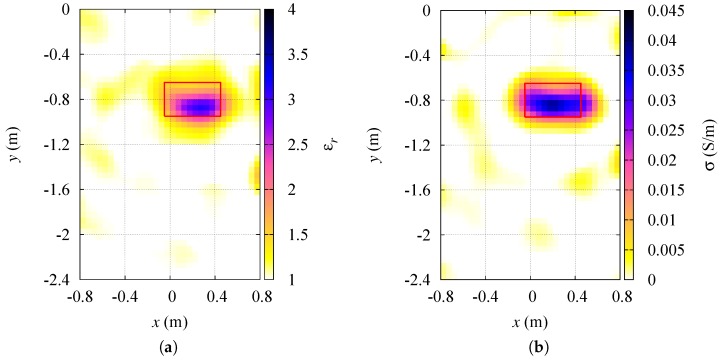
Aligned sensors. Reconstructed distributions of the (**a**) relative dielectric permittivity, and (**b**) electric conductivity of the region under test.

The result of the dielectric characterization of the target under test in this first scenario is reported in Figure 4, where the reconstructed distributions of both the relative dielectric permittivity and the electric conductivity are shown. A red line represents the contour of the actual target profile. The cylindrical target has been detected inside the inspection domain I and correctly characterized. However, a slight overestimation of its dielectric properties can be noticed close to the target center. Some artifacts, characterized by low values of relative permittivity and conductivity, also emerge in the background region, and can be attributed to the reduced number of adopted transceiving sensors. Table 2 reports the relative errors on the dielectric characterization of the targets, including also the results for p=2, i.e., the standard conjugate gradient method in Hilbert spaces, which provides higher characterization errors in the inspection domain.

#### 3.1.2. Non-Aligned Sensors

**Figure 5:**
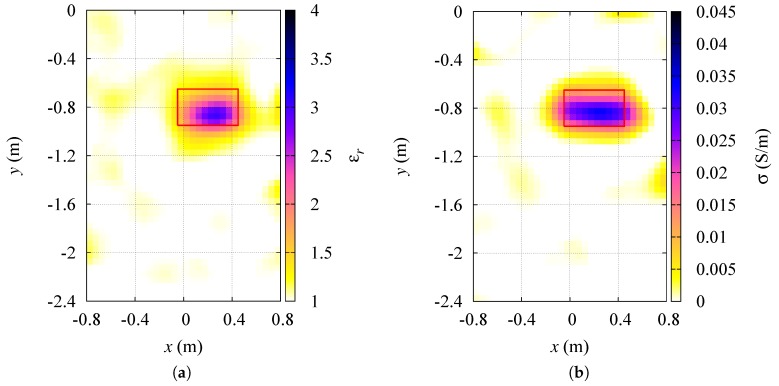
Non-aligned sensors. Reconstructed distributions of the (**a**) relative dielectric permittivity, and (**b**) electric conductivity of the region under test.

In the second simulated test case, a network of N=15 non-aligned sensors has been considered. Like in the previous configuration, transceiving elements are located around the inspection domain I, but this time their positions are not aligned with respect to *x* and *y* axes, and their separation is not uniform. In particular, their locations on the xy plane have been obtained by adding a random perturbation (with a maximum displacement of 20 cm) to the positions adopted in the first case, and are shown in Table 3. All the remaining parameters of the configuration, the target, and the inversion method remain the same as in the first case. Figure 5 shows the distributions of the reconstructed dielectric properties of the targets. It can be noticed that the reconstructed images look quite similar to those obtained with aligned microwave transceivers. The characterization errors, reported in Table 2, confirm the previously observed trends, and are comparable (slightly lower) to the case of aligned sensors.

#### 3.1.3. Multiple Targets in the Investigation Area

In a third case, the presence of two distinct objects inside the imaging area has been investigated. The same conditions of the first case have been considered, but the first target is now centered at $r_{c}=\left(-0.2,-0.8\right)\;m$, and a circular object has been added. The second dielectric target, which simulates a plastic rod, is centered at $r_{c}=\left(0.4,-1.5\right)\;m$ and has the following properties:

- radius r=0.2 m;
- relative dielectric permittivity $\epsilon_{r}=2$;
- electric conductivity σ=0.005 S/m.

**Figure 6:**
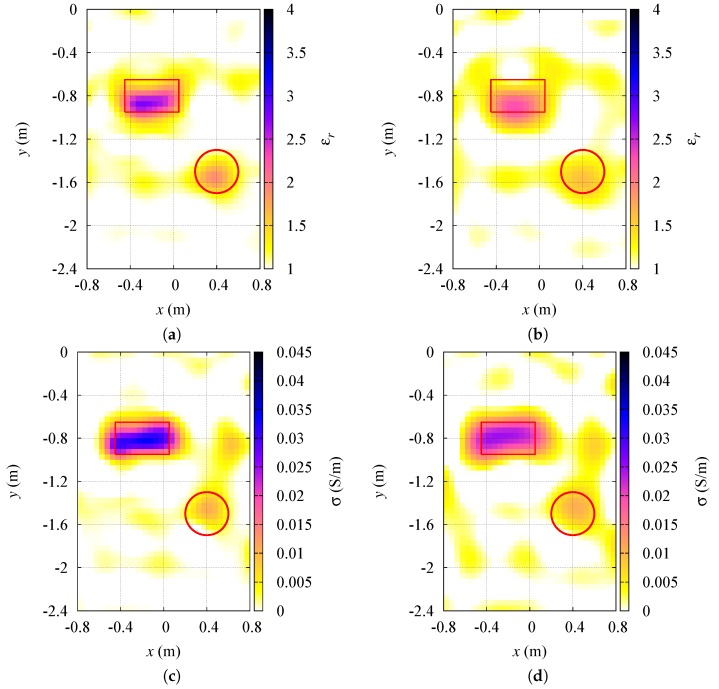
Multiple targets in the investigation area. Reconstructed distributions of the dielectric properties inside the investigation region: (**a**) relative dielectric permittivity, p=1.4; (**b**) relative dielectric permittivity, p=2; (**c**) electric conductivity, p=1.4; (**d**) electric conductivity, p=2.

In Figure 6 the obtained results are shown. For comparison purposes, in the same figure, the results achieved by using the usual inversion in a Hilbert space $L^{2}$ are also reported. The corresponding error parameters on the dielectric characterization can be found again in Table 2.

#### 3.1.4. Variation of the Number of Sensors

**Figure 7:**
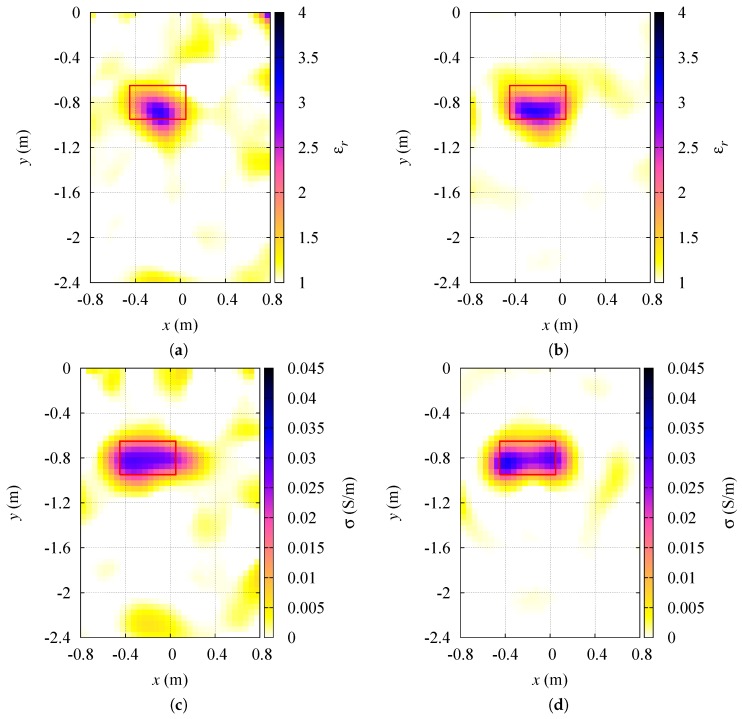
Variation of the number of sensors. Reconstructed distributions of the dielectric properties inside the investigation region: (**a**) relative dielectric permittivity, N=9; (**b**) relative dielectric permittivity, N=54; (**c**) electric conductivity, N=9; (**d**) electric conductivity, N=54.

Furthermore, the effect of changing the number of sensors has been studied, keeping all the other parameters like in Section 3.1.1. The same target located at $r_{c}=\left(-0.2,-0.8\right)\;m$ inside the inspection area I has also been considered. The sensing elements are aligned and equally spaced on the same three line segments as in Section 3.1.1. However, the number of sensors on each segment has been varied between 3 and 18 (obtaining an overall number of sensors $N\in \left[9,54\right]$). Figure 7 reports the reconstructed dielectric properties of the target for the cases with the minimum and the maximum number of sensors, i.e., respectively, N=9 and N=54. A number of elements below 9 did not give satisfactory results, while using more than 54 sensors did not produce substantial improvements. As for the reconstruction errors, they are shown in Table 4.

#### 3.1.5. Effect of Uncertainties in Sensor Positions

The effect of an uncertainty in sensor positions on the target characterization has also been assessed. In particular, like in the first test configuration, N=15 sensors are located on three lines outside I with y=0.5m, x=−1m, and x=1m, and their actual positions in the xy plane are reported in Table 1. To evaluate the behavior of the reconstruction procedure when the exact sensor positions are not known, the coordinates of sensors given to the inversion method have been perturbed with a displacement uniformly distributed in an interval of width $d\in \left[0.025,0.175\right]\;m$ centered at the actual sensor positions. In other words, inside the inversion procedure, the position of the *n*-th sensor is given by

(14) $$r_{n}^{inv}=r_{n}+u,\;\;n=1,\;\ldots ,\;N,$$

where u is a vector of two independent and identically distributed random variables with zero mean value and uniform distribution in the interval $\left[-d/2,d/2\right]$. The target is a single dielectric cylinder with rectangular cross section centered at $r_{c}=\left(-0.2,-0.8\right)\;m$ and characterized by:

- side lengths $s_{x}=0.5\;m$ and $s_{y}=0.3\;m$;
- relative dielectric permittivity $\epsilon_{r}=3$;
- electric conductivity σ=0.01 S/m.

**Figure 8:**
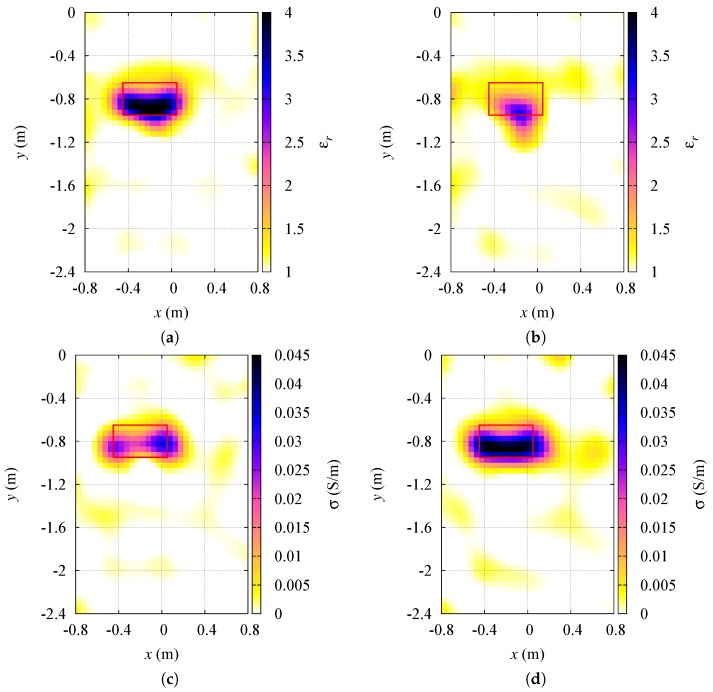
Effect of uncertainties in sensor positions. Reconstructed distributions of the dielectric properties inside the investigation region: (**a**) relative dielectric permittivity, d=0.025 m; (**b**) relative dielectric permittivity, d=0.125 m; (**c**) electric conductivity, d=0.025 m; (**d**) electric conductivity, d=0.125 m.

All the other parameters are kept the same as in the previous cases. The average relative errors on the dielectric characterization of targets are given in Table 5, whereas some examples of the reconstructed distributions dielectric properties of the inspection domain I are shown in Figure 8. As expected, the bigger is the sensors displacement with respect to the actual locations, the greater are characterization errors. However, looking at reconstruction results of Figure 8, it is evident that a rough detection of the target is possible even though the precise sensor locations are not available to the inversion method.

#### 3.1.6. Variation of the Dielectric Properties

The reconstruction capabilities of the proposed approach have also been evaluated with respect to the dielectric properties of the taget, which are the unknowns of the inverse problem. In particular, keeping all the other configuration parameters as in Section 3.1.1, we considered a circular dielectric cylinder centered at $r_{c}=\left(-0.5,-1\right)$ m. The target is characterized by:

- radius r=0.125 m;
- relative dielectric permittivity varied in the interval $\epsilon_{r}\in \left[1.5,80\right]$;
- electric conductivity σ=0.01 S/m.

**Figure 9:**
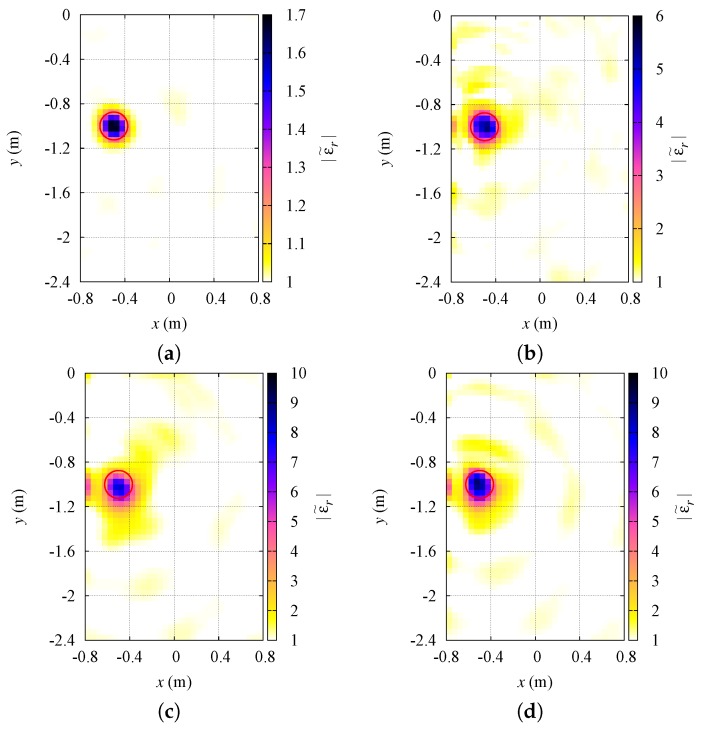
Variation of the dielectric properties. Reconstructed distributions of the magnitude of the complex dielectric permittivity inside the investigation region: (**a**) $\epsilon_{r}=1.5$; (**b**) $\epsilon_{r}=6$; (**c**) $\epsilon_{r}=10$; (**d**) $\epsilon_{r}=50$.

Some examples of the obtained results are shown in Figure 9, where the reconstructed magnitude of the complex dielectric permittivity has been reported in different cases. It has been found that for $\epsilon_{r}\leq 10$ the magnitude of the complex dielectric permittivity is correctly retrieved (Figure 9a–c). When the permittivity is higher, the quantitative reconstruction is not accurate, but a quite good qualitative localization of the cylinder is still possible (Figure 9d).

#### 3.1.7. Spatial Resolution

The resolution of the inversion method has been analyzed with respect to the wavelength and the number of sensors. In particular, we analyzed the minimum spacing between the external boundaries of two cylinders which allows to resolve the objects as separated.

The investigation area I has been chosen equal to that of Section 3.1.1, with *N* aligned sensors located on the same line segments. Two dielectric cylinders with circular cross section have been considered, with radius r=λ/8 (λ being the wavelength) and characterized by a relative dielectric permittivity $\epsilon_{r}=2$ and electric conductivity σ=0.01 S/m. The first target is located at $r_{c1}=\left(-\lambda /2,-\lambda \right)$, and the second one at $r_{c2}=\left(-\lambda /2,-\lambda +d+2r\right)$, so that the separation between their boundaries is equal to *d*. The distance *d* has been varied between 0 and 2λ/3. The resolution has been studied by analyzing the ratio between the reconstructed contrast function magnitude in the centers of the cylinders and in the middle point between cylinder centers. When the amplitude ratio is higher than unity, it means that a single target is reconstructed instead of two distinct cylinders.

**Figure 10:**
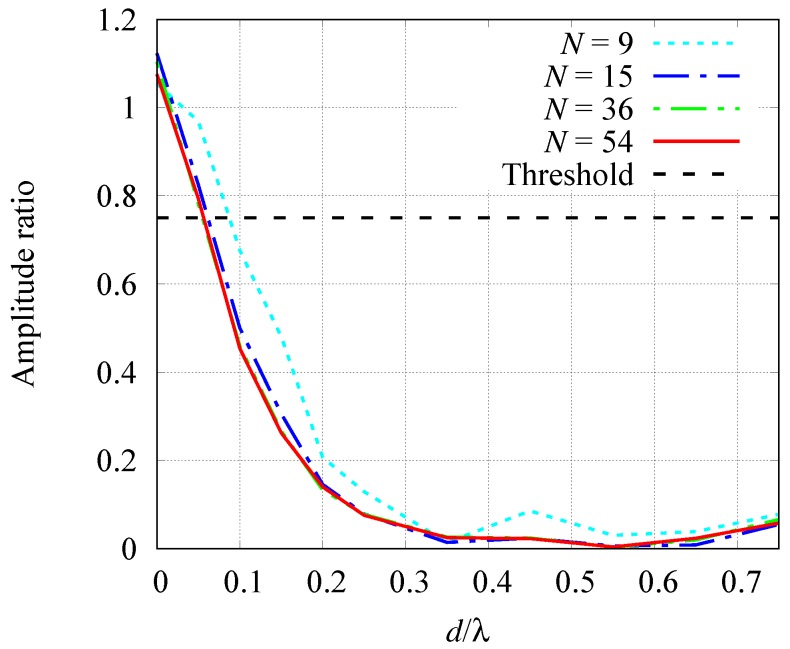
Spatial resolution. The separation distance between the two cylinders is shown on the abscissa, while on the ordinate axis is the ratio between the amplitudes. Light blue line: 9 sensors; blue line: 15 sensors; green line 36 sensors; red line: 54 sensors.

The resulting amplitude ratio is reported in Figure 10 versus the separation *d*. Four cases with different number of sensors *N* have been considered. With a threshold of 75%, it appears that the minimum separation for resolving the two cylinders is close to λ/10 (the corresponding distance between $r_{c1}$ and $r_{c2}$ is about λ/3). As for the number of sensors, results show that for N≥15 the resolution is almost the same in any case, while, in the case N=9 a slight deterioration of the results is noticeable. When the number of sensors is N<9 not only the resolution, but the overall result of the reconstruction also quickly deteriorates.

It is worth noting that all the involved quantities have been expressed in terms of the wavelength. Therefore, results are independent from the operating frequency (with a proper scaling of the target properties and configuration parameters).

### 3.2. Mapping from Experimental Data

Experimental data on the test cases considered for the simulations were not available at the time of writing this work. However, in order to obtain preliminary information on using the method in realistic cases, measurements from the Fresnel Institute database [38], which is a de-facto standard reference for testing inversion method against experimental data, have been used. However, in order to make a fair comparison, we adapted the investigation area to obtain the same dimensions, with respect to the wavelength, used in the simulated data. In particular, the measurements related to the *dielTM* object have been considered. This object is a single plastic rod with the following properties:

- radius r=0.015 m;
- relative dielectric permittivity $\epsilon_{r}=3\pm 0.3$;
- electric conductivity σ≃0 S/m.

Data were obtained in a controlled environment inside an anechoic chamber. The antenna sensor used for collecting the data was a double-ridged horn operating from 1 GHz to 18 GHz. More details about the measurement set-up can be found in [38] and the related papers.

**Figure 11:**
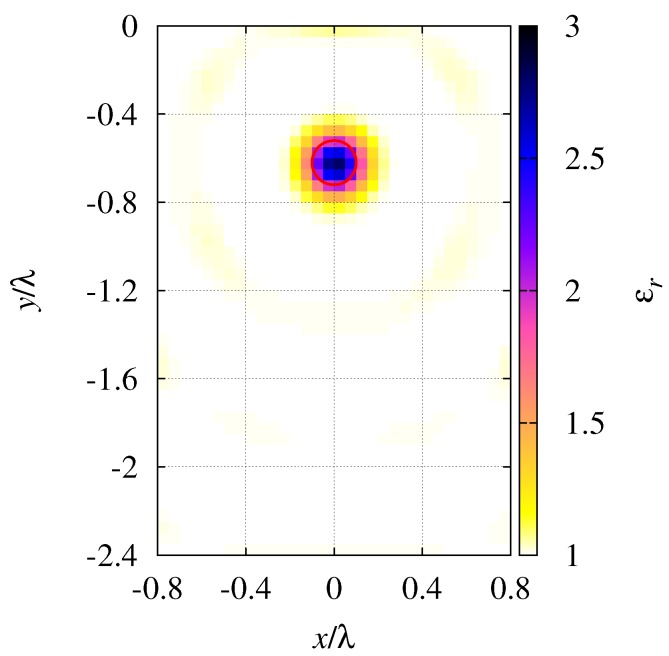
Mapping from experimental data. Reconstructed distributions of the relative dielectric permittivity of the region under test.

The reconstruction has been performed at the frequency f=2GHz. Table 6 reports the reconstruction errors, while a picture of the reconstructed inspection domain is shown in Figure 11. In order to facilitate a comparison with the previous simulations, dimensions are scaled according to the wavelength. As can be noticed, even in this first experimental case, the target characterization is quite accurate.

## 4. Conclusions

The nowadays pervasive use of wireless sensor networks is continuously stimulating new and challenging applications. Among them, the possibility of characterizing dielectric targets could look still visionary today, but it is certainly promising for a near future. In principle, the use of a sensor network could not only allow the mapping of the dielectric characteristics in static settings, but could be useful even in the case of moving sensors, e.g., those installed on board in robots and drones. However, even under simplifying assumptions, the problem is not easy to solve. In this contribution, a Newton-conjugate-gradient algorithm is preliminary assessed to provide a full dielectric characterization of an unknown region of space, surrounded by a network of microwave elements acting as transceivers. Basically, this characterization method operates a reconstruction of the dielectric properties of the targets by processing the scattered electric field measured by the sensors and solving a nonlinear and ill-posed inverse problem. Some initial results, mainly obtained in a simulated environment, have been shown in order to obtain a first proof-of-concept of the proposed approach. A preliminary experimental test case has also been considered. Clearly, there are several points that inspire further developments, including the method validation in more realistic conditions and with a comprehensive set of experimental data, the application of variable-exponent techniques in Lebesgue spaces [46], as well as the extension to amplitude-only inverse-scattering methods, which seem particularly suitable in real-word applications.

## Figures and Tables

**Table 1:** Positions of the Microwave Sensors in the First Simulated Scenario. Aligned sensors.

Sensor ID	*x* (m)	*y* (m)	Sensor ID	*x* (m)	*y* (m)	Sensor ID	*x* (m)	*y* (m)
1	−1.00	0.50	6	−1.00	−2.00	11	1.00	−2.00
2	−0.50	0.50	7	−1.00	−1.50	12	1.00	−1.50
3	0	0.50	8	−1.00	−1.00	13	1.00	−1.00
4	0.50	0.50	9	−1.00	−0.50	14	1.00	−0.50
5	1.00	0.50	10	−1.00	0	15	1.00	0

**Table 2:** Relative Errors on the Dielectric Characterization of the Targets in Cases of Aligned Sensors, Non-Aligned Sensors and Multiple Targets in the Investigation Area.

Test Case	Value of *p*	Relative Characterization Errors		
		Background Area, $\Gamma_{b}$	Target Area, $\Gamma_{t}$	Whole Inspection Domain, $\Gamma_{I}$
Aligned sensors	1.4	0.122	0.456	0.135
	2.0	0.205	0.453	0.215
Non-aligned sensors	1.4	0.107	0.431	0.120
	2.0	0.182	0.439	0.192
Multiple targets	1.4	0.142	0.369	0.158
	2.0	0.216	0.394	0.229

**Table 3:** Positions of the Microwave Sensors in the Second Simulated Scenario.

Sensor ID	*x* (m)	*y* (m)	Sensor ID	*x* (m)	*y* (m)	Sensor ID	*x* (m)	*y* (m)
1	−1.05	0.40	6	−0.90	−2.10	11	0.90	−2.00
2	−0.50	0.50	7	−1.00	−1.40	12	1.00	−1.60
3	0.10	0.45	8	−0.95	−1.10	13	1.10	−1.20
4	0.50	0.55	9	−0.85	−0.50	14	0.85	−0.60
5	1.10	0.45	10	−1.00	0.10	15	0.95	0.20

**Table 4:** Relative Errors on the Dielectric Characterization Versus the Number of Sensors.

Number of Sensors	Relative Characterization Errors		
	Background Area, $\Gamma_{b}$	Target Area, $\Gamma_{t}$	Whole Inspection Domain, $\Gamma_{I}$
9	0.177	0.448	0.166
18	0.115	0.445	0.101
27	0.120	0.418	0.108
36	0.112	0.391	0.101
45	0.115	0.390	0.104
54	0.110	0.380	0.099

**Table 5:** Relative Errors on the Dielectric Characterization Versus the Sensor Displacement Width.

Displacement width, *d* (m)	Relative Characterization Errors		
	Background Area, $\Gamma_{b}$	Target Area, $\Gamma_{t}$	Whole Inspection Domain, $\Gamma_{I}$
0.025	0.145	0.347	0.153
0.075	0.172	0.524	0.186
0.125	0.175	0.626	0.192
0.175	0.161	1.060	0.196

**Table 6:** Relative Errors on the Dielectric Characterization from Experimental Data.

Relative Characterization Errors		
**Background area, $\Gamma_{b}$**	**Target area, $\Gamma_{t}$**	**Whole inspection domain, $\Gamma_{I}$**
0.0297	0.255	0.0276
